# A Comprehensive Pan-Cancer Analysis Revealing IGF2 Gene as a Diagnostic and Prognostic Biomarker

**DOI:** 10.7759/cureus.110688

**Published:** 2026-06-11

**Authors:** Wala Abdallah, Ayman E Abbas, Amal H. A Assed, Hiba Elrashid Yagoub, Samar Doleeb, Alaa Abdalla, Ahmed Hamid, Mohamed Alfaki

**Affiliations:** 1 Microbiology, Faculty of Medical Laboratory Science, Nile University, Khartoum, SDN; 2 Plastic Surgery, Burjeel Medical City, Abu Dhabi, ARE; 3 Endemic Diseases, University of Khartoum, Khartoum, SDN; 4 Oncology, National Cancer Institute, University of Gezira, Wad Madani, SDN; 5 Biotechnology, Fergusson College (Autonomous), Pune, IND; 6 Cardiology, Salam Centre for Cardiac Surgery, Khartoum, SDN; 7 College of Pharmacy, National Ribat University, Khartoum, SDN; 8 Computer Science, Al-Neelain University, Khartoum, SDN

**Keywords:** diagnostic biomarker, expression analysis, igf2, pan-cancer analysis, prognostic biomarker

## Abstract

Background: Cancer, one of the leading causes of mortality worldwide, is driven by genetic alterations promoting uncontrolled cell growth and metastasis. Among these genetic players, the insulin-like growth factor 2 (IGF2) gene has emerged as a significant factor in tumorigenesis. IGF2, a growth factor primarily produced in the liver, interacts with insulin and IGF receptors, influencing cell proliferation and survival. IGF2 is a known driver in fetal development and specific hepatic malignancies; its multi-omic diagnostic, prognostic, and immunological landscapes across diverse tissue barriers remain unmapped. This study presents a comprehensive pan-cancer analysis to systematically define the biomarker potential and epigenetic regulation of IGF2 across multiple human cancers.

Methods: We utilized various bioinformatic platforms, including Tumor Immune Estimation Resource (TIMER), Gene Expression Profiling Interactive Analysis (GEPIA), UALCAN, and cBioPortal, to assess IGF2 gene expression and mutation profiles. Immune infiltration analysis evaluated IGF2's role in tumor-immune interactions.

Gene expression data from the Cancer Genome Atlas (TCGA) and Genotype-Tissue Expression (GTEx) databases were analyzed, and findings were validated using the Gene Expression Omnibus (GEO). Kaplan-Meier survival analysis was applied to investigate the correlation between IGF2 expression and overall survival in different cancers.

Results: IGF2 was significantly upregulated in cholangiocarcinoma (CHOL), liver hepatocellular carcinoma (LIHC), kidney chromophobe (KICH), and stomach adenocarcinoma (STAD) with P-values of 3.72E-05, 3.30-E08, 1.60E-03, and 1.43E-04, respectively. Survival analysis revealed that elevated IGF2 expression was associated with good prognosis in kidney renal papillary cell carcinoma (KIRP) (P = 7.2E-05). Immune infiltration analysis demonstrated a significant correlation between IGF2 expression and the presence of macrophages and CD4+ T cells in STAD (P = 1.30E-10, 2.61E-06, respectively), suggesting a role in modulating the tumor immune microenvironment. Genetic alterations in IGF2 were observed in multiple cancers, with missense mutations and deep deletions being the most prevalent. Patients with IGF2 mutations showed a trend toward poorer survival outcomes compared to those without mutations; however, the difference was not statistically significant (P = 0.384).

Conclusion: This study revealed that IGF2 plays a potential role as a diagnostic biomarker for CHOL, KICH, LIHC, and STAD and a prognostic biomarker role in KIRP.

## Introduction

Cancer is characterized by an unregulated proliferation of cells, which possess the ability to disseminate to various anatomical sites as metastases. This pathological condition not only compromises physical health but also detrimentally impacts mental well-being and economic stability. The global financial burden of cancer exceeds one trillion dollars annually [1]. Cancer remains one of the predominant etiologies of mortality, with an annual incidence exceeding 10 million deaths [2]. Throughout the process of carcinogenesis, specific genes may become upregulated, thereby facilitating cellular proliferation or inhibiting apoptotic mechanisms, while other genes that are ordinarily tasked with tumor suppression may lose their functional integrity, culminating in the development of cancer. The inactivation of tumor-suppressing genes may occur through mutations, complete gene deletion, or epigenetic silencing [3]. The identification of precise biomarkers is essential for the accurate diagnosis and effective treatment of cancer [4]. The principal risk factors associated with cancer development encompass tobacco use, excessive alcohol intake, obesity, sedentary lifestyles, infectious agents, and ultraviolet radiation exposure. Within most affluent nations, the cancer types most frequently diagnosed in adults include lung, colorectal, breast, cutaneous melanoma, and prostate cancers [5].

Insulin-like growth factors (IGFs) are part of a complex system that all work together to influence growth. This system consists of insulin, insulin receptor (IR), insulin-like growth factor (IGF I), IGF II, IGF receptor, and insulin-like growth factor binding protein (IGFBP), insulin-like growth factor 2 (IGF2) mainly produced in liver and tissues, where it has both autocrine and paracrine effects. The IGF2 gene is located near the insulin gene on chromosome 11p15.5 and encodes a mature 67 amino acid (7.5 kDa) peptide [6]. IGF2 is a growth factor that has structural and regulatory roles, which exhibit pleiotropic and tissue-specific functions [7]. Both the IR and the insulin-like growth factor 1 receptor (IGF-IR), which belong to the tyrosine kinase family, can bind to insulin, IGF-1, and IGF-2, but with varying degrees of affinity. The IR is encoded by the INSR gene, located on chromosome 19p13.2. The coding region includes 22 exons; splicing of exon 11 generates two structurally different isoforms: IR-A (expressed in fetal tissues, central nervous system (CNS), hematopoietic cells, and cancer cells) and IR-B (expressed in the major insulin target tissues). IR-A exhibits high affinity binding for Insulin and IGF-2 while demonstrating low affinity binding for IGF-1. Conversely, IR-B displays high affinity binding for insulin, but low affinity binding for both IGF-1 and IGF-2 [8]. Activation of IR-A by insulin mainly leads to metabolic effects, while activation of IR-A by IGF-II triggers mitogenic effects. These distinct outcomes are linked to different intracellular pathways. IR-A is more expressed in fetal cells and certain tumors, suggesting its role in both fetal development and cancer. IGF-II interacts with both IGF-I-R and IR-A, highlighting its potential significance in growth and cancer [9].

While IGF2 is a known driver in fetal development and specific hepatic malignancies, its multi-omic diagnostic, prognostic, and immunological landscapes across diverse tissue barriers remain unmapped. Therefore, this study presents a comprehensive pan-cancer analysis to systematically define the biomarker potential and epigenetic regulation of IGF2 across multiple human cancers.

## Materials and methods

Knowledge gap assessment

The gene was searched in NCBI gene databases, then searched in PubMed, and all publications that include IGF2 literature were collected. The query was generated using Biofix Tool (https://mohamedalfaki.shinyapps.io/BioFixV5/), which includes all official names, symbols, and Aliases of the gene [10]. The (tw = text words) was used to limit the search to text found in the title and abstract. (IGF2[tw] OR "insulin like growth factor 2"[tw] OR "T3M-11-derived growth factor"[tw] OR "insulin-like growth factor 2 (somatomedin A)"[tw] OR "insulin-like growth factor II"[tw] OR "insulin-like growth factor type 2"[tw] OR "preptin"[tw] OR "C11orf43"[tw] OR "GRDF"[tw] OR "IGF-II"[tw] OR "PP9974"[tw] OR "SRS3[tw]") AND "cancer" AND GEPIA[tw]”

Gene expression profile

TIMER Database

Tumor Immune Estimation Resource (TIMER) is a web-based computational tool that provides a comprehensive analysis of gene expression data to estimate the levels of tumor-infiltrating immune cells "TIICs” [11]. TIMER depends on data from the Cancer Genome Atlas (TCGA), including 20,000 samples across 33 cancer types. TIMER contains different parameters. We used Diff Exp analysis to investigate the differential expression of the IGF2 gene in tumors versus normal tissues across various cancer types, indicating whether it was upregulated or downregulated.

GEPIA Database

Gene Expression Profiling Interactive Analysis (GEPIA) is a web-based tool designed for analyzing gene expression data derived from the TCGA and GTEx (Genotype-Tissue Expression) databases [12]. GEPIA has different parameters: differential expression analysis, survival analysis, profiling, and correlation. We used Expression DIY and then a box plot to see the expression of the IGF2 gene across different cancers.

UALCAN Database Analysis

The University of Alabama at Birmingham Cancer data analysis portal (UALCAN, https://ualcan.path.uab.edu/) is a comprehensive web tool for analyzing gene expression, allowing for comparisons between tumor and normal samples, as well as within tumor populations based on variables like cancer stage, patient age, tumor grade, and other clinical characteristics [13]. In this study, we use it to determine the correlation between the IGF2 gene and clinical pathological conditions such as cancer stage, age, race, and gender. Additionally, we used it to validate the expression analysis of the gene from both the GEPIA and TIMER databases and to examine the DNA methylation status of IGF2.

Immune Cell Infiltration Analysis

TIMER was used to study the correlation between IGF2 expression and immune infiltration of CD4+ T cells, macrophages, neutrophils, and dendritic cells, and the correlation between immune infiltration and prognosis of the tumor.

Overall Survival

Overall survival is a crucial endpoint used to measure the efficacy of cancer treatment. In this study, we used GEPIA and Kaplan-Meier (KM) plotter to determine the association between IGF2 expression and the prognosis of cancer. For GEPIA, we used survival analysis, which included hazard ratio, cutoff point (50%), confidence interval (95%), and corresponding P-value. The KM plotter (https://kmplot.com/analysis/) is a comprehensive database that assesses the relationships between the expression of all major biomolecule classes (mRNA, miRNA, protein, and DNA) and key survival biomarkers in a collection of over 35,000 samples spanning 21 distinct tumor types [14].

Genetic Alteration Analysis

cBioPortal for cancer genomics database is a comprehensive web tool that enables the investigation, visualization, and analysis of multi-faceted cancer genomics data [15]. The platform includes four interconnected views: study the interactive exploration of genomics and clinical data in a dataset, results for the detailed analysis and visualization of genomics alterations in specific genes, comparison of genomics and clinical features in two or more cohorts, and visualization and interpretation of genomic and clinical data in a patient [16]. In this study, we used the cBioPortal to see the genetic alteration of the IGF2 gene and other types of mutation across different cancers.

Gene Correlation and Enrichment Analysis

The University of Alabama at Birmingham Cancer Data Analysis Portal (UALCAN) was used to analyze IGF2-correlated genes across different cancer types, based on gene expression data from three databases: TIMER, GEPIA, and UALCAN. The correlation parameter is utilized to identify these gene associations. The resulting gene list is then analyzed using the STRING database (https://string-db.org/), a platform for protein-protein/gene interaction (PPI) analysis that integrates both physical interactions and functional associations among proteins/genes [17]. Additionally, we used the GeneMANIA database (http://genemania.org/) to develop the gene network of IGF2-correlated genes and predicted potential target genes. GeneMANIA database is a versatile, user-friendly platform that helps generate insights about gene functions, analyze gene groups, and identify key genes for further functional experiments [18]. To understand the underlying biological processes in different cancers, we performed enrichment analysis for genes associated with each cancer type (CHOL, KICH, LIHC) separately. This analysis focused on identifying enriched pathways in the KEGG database and functional categories within Gene Ontology (GO), including the biological process, cellular component, and molecular function. We used Enrichr (https://maayanlab.cloud/Enrichr/), a user-friendly web tool that provides clear visualizations, to perform the analysis for each cancer type individually. By comparing the results across all three cancers, we aimed to identify shared pathways and functions potentially involved in common mechanisms underlying these cancers [19].

Validation

To confirm our findings from the gene expression analysis, we utilized the Gene Expression Omnibus (GEO), a resource from the National Center for Biotechnology Information (NCBI). Established in 2000, GEO is an international public repository designed to archive and openly share high-throughput gene expression and other functional genomics data. It is widely used by researchers for transcriptomic studies [20]. We leveraged GEO to verify IGF2 gene expression across four types of cancers: CHOL, KICH, LIHC, and STAD. Relevant studies were identified, and GEO2R was employed to analyze the data by comparing different groups of samples, focusing on the differences between normal and tumor tissues. The results from the GEO database were then uploaded to the Julius AI platform (https://julius.ai/), a tool designed to streamline data analysis and visualization in scientific research. The data was visualized using a volcano plot, which provides a clear representation of significant patterns, supporting our analysis as part of our research method.

## Results

**Figure 1:**
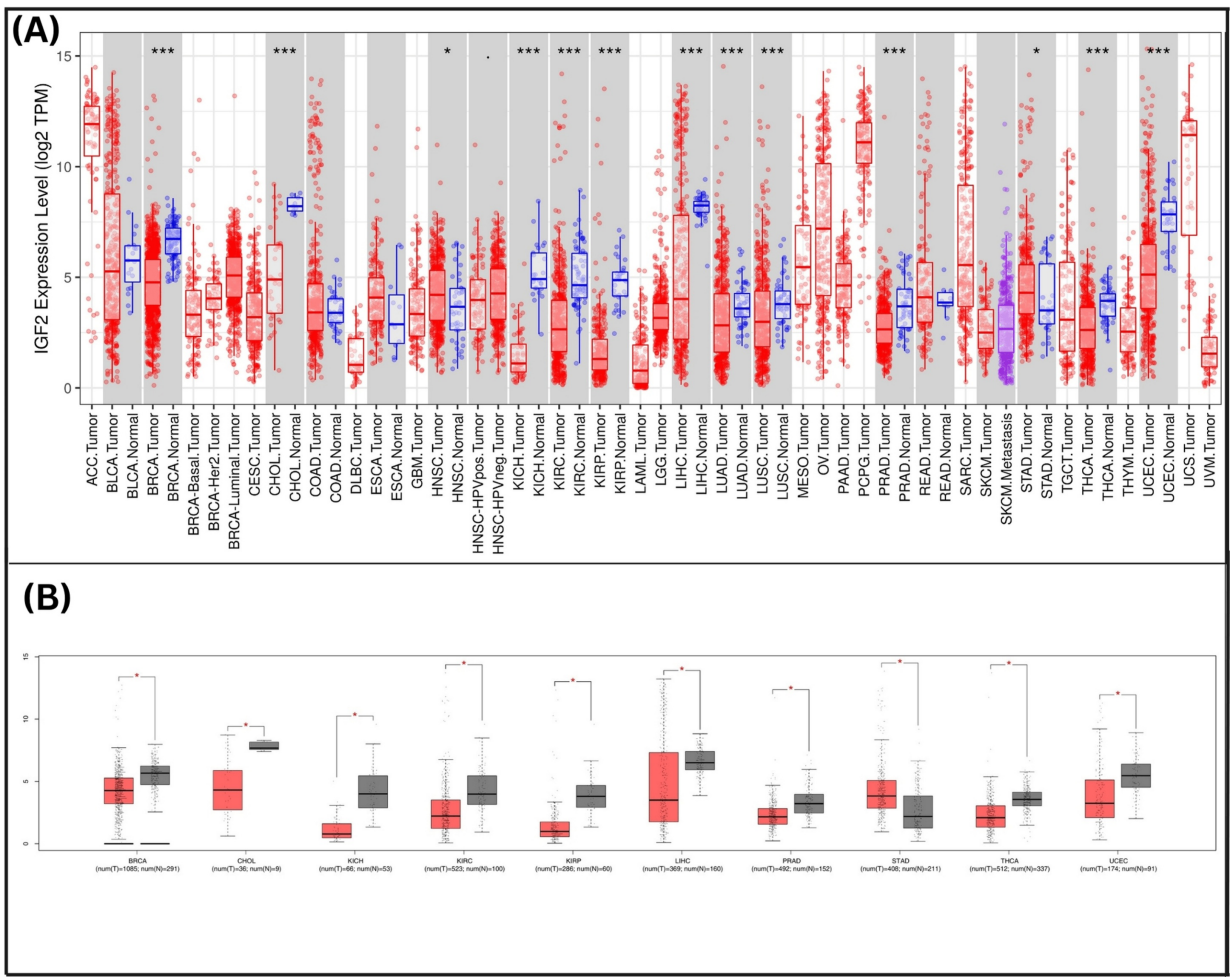
IGF2 expression profile across different cancer types using the TIMER and GEPIA databases (P-values: * < 0.05; ** <0.01; *** <0.001). (A): IGF2 expression using the TIMER database (B): IGF2 expression using the GEPIA database (N): Normal; (T): Tumor; TIMER: Tumor Immune Estimation Resource; GEPIA: Gene Expression Profiling Interactive Analysis; IGF2: Insulin-Like Growth Factor 2

In this study, we performed a pan-cancer analysis to assess the diagnostic and prognostic role of IGF2 across various cancer types using TIMER, GEPIA, and UALCAN. The IGF2 gene was found to be highly expressed in cancer types commonly identified in both TIMER and GEPIA, including breast carcinoma (BRCA), cholangiocarcinoma (CHOL), kidney chromophobe (KICH), kidney renal clear cell carcinoma (KIRC), kidney renal papillary cell carcinoma (KIRP), liver hepatocellular carcinoma (LIHC), prostate adenocarcinoma (PRAD), stomach adenocarcinoma (STAD), thyroid carcinoma (THCA), and uterine corpus endometrial carcinoma (UCEC), as shown in Figures 1A, 1B. 

Table 1 presents the expression values and sample numbers obtained from GEPIA.

**Table 1:** Summary of tumor and normal sample composition across cancer cohorts (GEPIA). KIRC: Kidney renal clear cell carcinoma; KIRP: kidney renal papillary cell carcinoma; CHOL: cholangiocarcinoma; BRCA: breast carcinoma; PRAD: prostate adenocarcinoma; LIHC: liver hepatocellular carcinoma; STAD: stomach adenocarcinoma; UCEC: uterine corpus endometrial carcinoma; THCA: thyroid carcinoma; KICH: kidney chromophobe

Rank	Cohort	Tumor Samples	Normal Samples	Tumor Percentage
1	KIRC	523	100	84.0%
2	KIRP	286	60	82.7%
3	CHOL	36	9	80.0%
4	BRCA	1,085	291	78.9%
5	PRAD	492	152	76.4%
6	LIHC	369	160	69.8%
7	STAD	408	211	65.9%
8	UCEC	174	91	65.7%
9	THCA	512	337	60.3%
10	KICH	66	53	55.5%

As shown in Table 1, KIRC had the highest proportion of tumor samples (84%), while KICH had the lowest (55.5%). Most of the remaining cohorts were in the 65-80% range, suggesting a clear tumor‑dominant composition overall.

**Figure 2:**
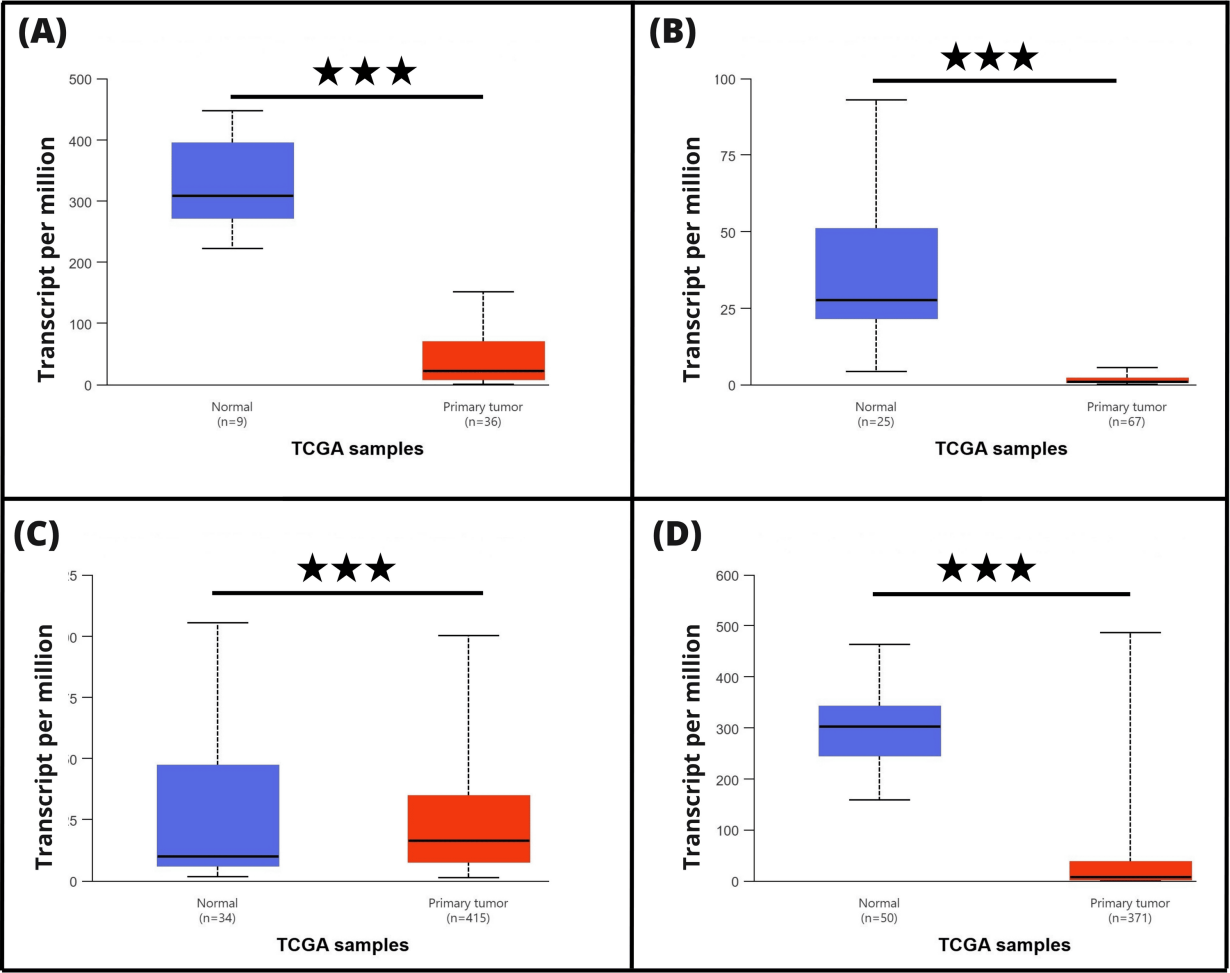
IGF2 expression profile across different cancer types using the UALCAN database (P-values: * < 0.05; ** <0.01; *** <0.001). (A): Expression of IGF2 in CHOL (B): Expression of IGF2 in KICH (C): Expression of IGF2 in STAD (D): Expression of IGF2 in LIHC CHOL: Cholangiocarcinoma; KICH: kidney chromophobe carcinoma; STAD: stomach adenocarcinoma; LIHC: liver hepatocellular carcinoma

The UALCAN database was utilized to assess IGF2 expression, thereby validating its expression patterns observed in the 10 cancer samples obtained from the TIMER and GEPIA databases (BRCA, CHOL, KICH, KIRC, KIRP, LIHC, PRAD, STAD, THCA, and UCEC). We found that IGF2 expression was significant in four types of cancers that were shared between the three databases: CHOL, KICH, STAD, and LIHC, as shown in Figure 2.

Clinical parameter analysis for IGF2 expression

We analyzed the correlation between IGF2 expression and various clinicopathological parameters, including age (young adults “21-40 years,” middle-aged adults “41-60 years”, older adults “61-80 years”, and elders “81-100 years”), gender (male, female), race (African American, Caucasian, and Asian), and cancer stages (stages 1, 2, 3, and 4) across four cancers (CHOL, KICH, LIHC, and STAD) that were consistent between the three databases TIMER, GEPIA and UALCAN. P-value thresholds of both 0.05 and 0.01 were used to determine statistical significance.

**Figure 3:**
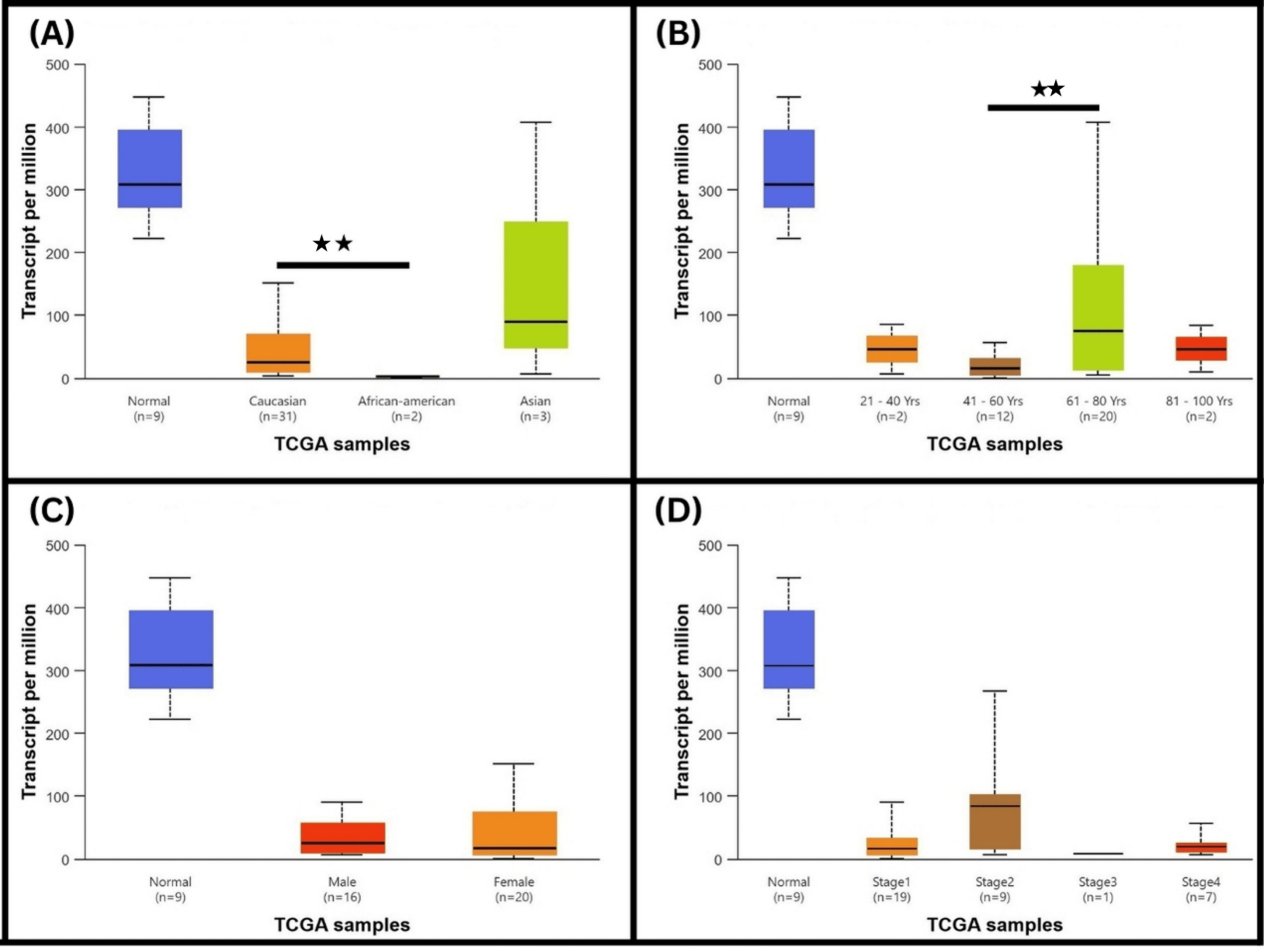
Expression of IGF2 in CHOL across different clinical parameters (race, age, gender and cancer stages) (P-values: * < 0.05, ** <0.01, *** <0.001). (A): Expression of IGF2 in CHOL based on patient's race (B): Expression of IGF2 in CHOL based on patient's age (C): Expression of IGF2 in CHOL based on patient's gender (D): Expression of IGF2 in CHOL based on individual cancer stages IGF2: Insulin-like growth factor 2; CHOL: cholangiocarcinoma

In CHOL, IGF2 expression showed significant differences based on race, with a notable distinction between Caucasians and African Americans (P = 1.12E-03), as shown in Figure 3A, though there were no significant differences between other ethnic groups. In terms of age, a significant difference was observed between the age “41-60” and age “61-80” groups (P = 6.72E-03), as shown inFigure 3B. However, there were no significant differences in IGF2 expression between male patients and female patients, nor across all stages of cancer, as in Figures 3C, 3D.

**Figure 4:**
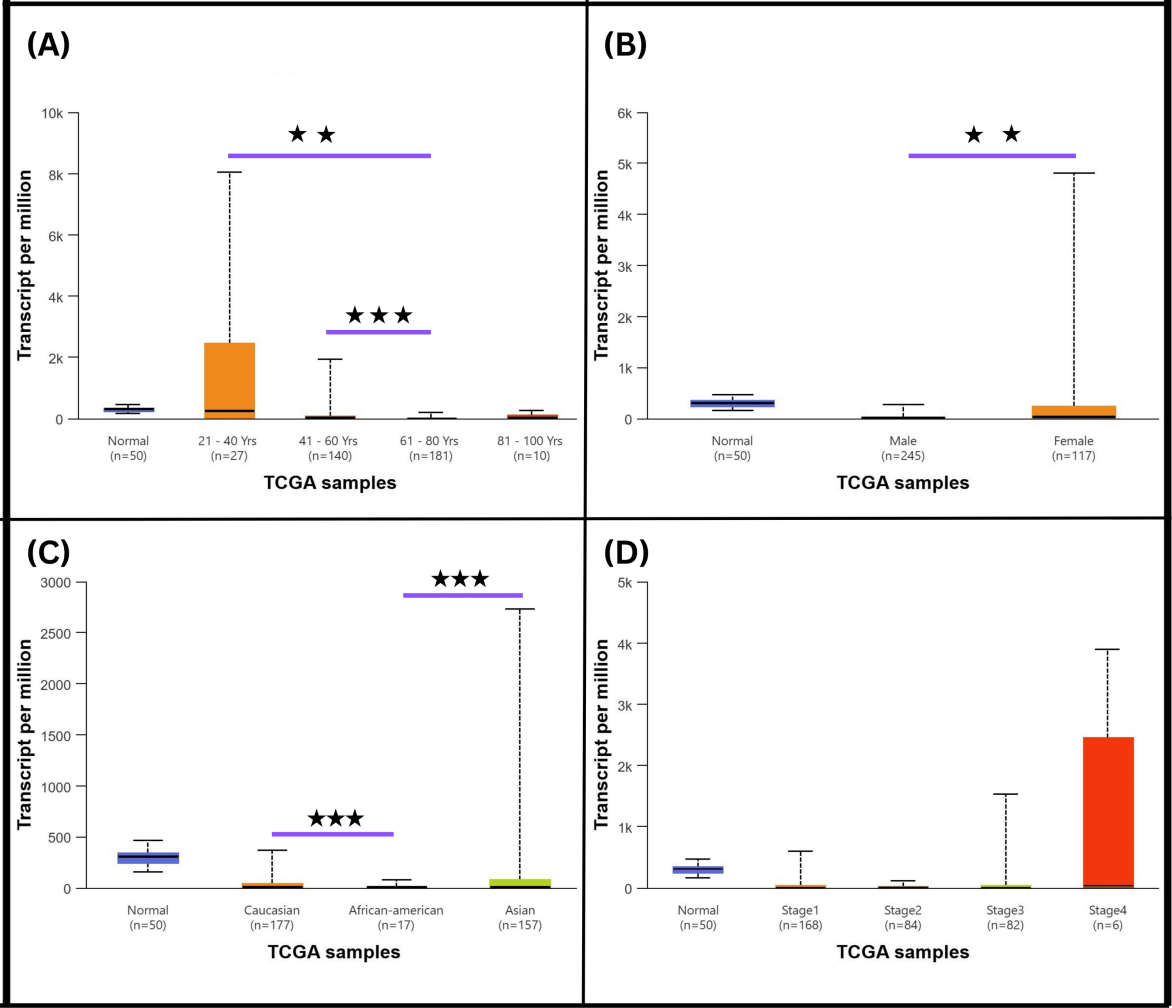
Expression of IGF2 in LIHC across different clinical parameters (age, gender, race and cancer stages) (P-values: * < 0.05, ** <0.01, *** <0.001). (A): Expression of IGF2 in LIHC based on patient's age (B): Expression of IGF2 in LIHC based on patient's gender (C): Expression of IGF2 in LIHC based on patient's race (D): Expression of IGF2 in LIHC based on individual cancer stages IGF2: Insulin-like growth factor 2; LIHC: liver hepatocellular carcinoma

InLIHC, when analyzing IGF2 expression concerning age, significant differences were observed between the age "21-40" and age "61-80" groups (P = 6.70E-03), as well as between the age "41-60" and age "61-80" groups (P = 6.39E-05). However, there was no significant difference between the age “21-40” and age “81-100” groups, as shown in Figure 4A. There was also a significant difference in IGF2 expression between male patients and female patients (P = 4.32E-03), as shown in Figure 4B.

IGF2 expression showed significant differences based on race, particularly between Caucasians and African Americans (P = 2.13E-06), as well as between African Americans and Asians (P = 8.27E-09), as shown in Figure 4C. Additionally, across all stages of liver cancer, no significant differences in IGF2 expression were detected, as shown in Figure 4D.

**Figure 5:**
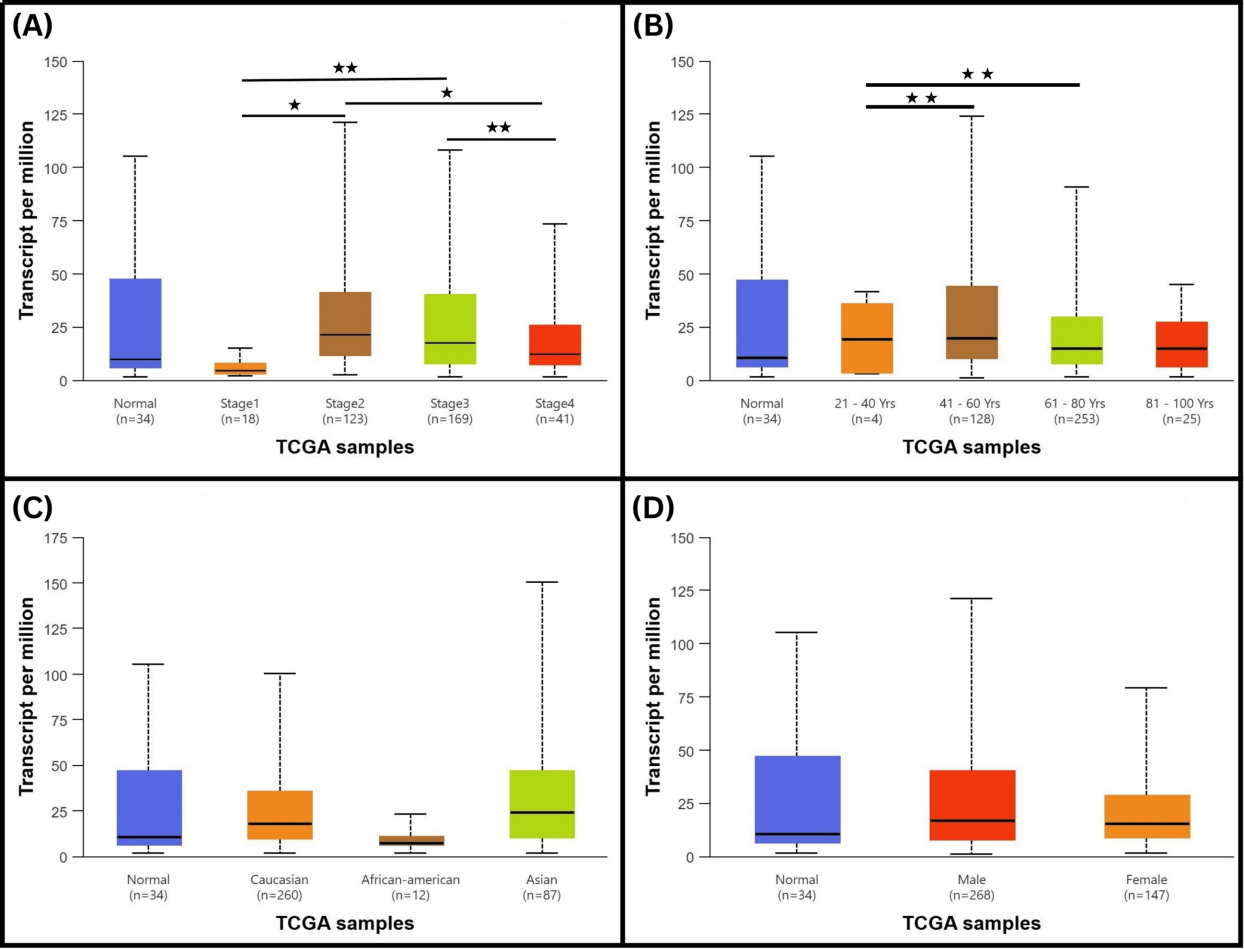
Expression of IGF2 in STAD cancer across different clinical parameters (age, gender, race, and cancer stages) (p-values: * < 0.05, ** <0.01, *** <0.001). (A): Expression of IGF2 in STAD based on individual cancer stages (B): Expression of IGF2 in STAD based on patient's age (C): Expression of IGF2 in STAD based on patient's race (D): Expression of IGF2 in STAD based on patient's gender IGF2: Insulin-like growth factor 2; STAD: stomach adenocarcinoma

For STAD, IGF2 expression showed significant variation when comparing cancer stages. Specifically, a significant difference was found between Stage1 and Stage2 (P=1.881920E-02), stage 1 and stage3 (P= 4.802000E-03), Stage2-vs-Stage4 (P=2.939800E-02), and between Stage3-vs-Stage4 (P= 6.399400E-03), as shown in Figure 5A. while no significant differences were observed between the other stages. Regarding age, IGF2 expression was significantly different between the age “21-40” and age “41-60” groups (P = 5.92E-03), as well as between the age “21-40” and age “61-81” groups (P = 6.98E-03), as shown inFigure 5B, though no significant differences were detected across other age groups. There were no significant differences in IGF2 expression between racial groups or between male patients and female patients in STAD (Figures 5C, 5D).

Inthe case of KICH, IGF2 expression did not show significant differences across racial groups, with no substantial variation observed between Caucasians, African Americans, and Asians. Similarly, there were no significant differences in IGF2 expression between males and females. When considering age, no significant differences were found among the various age groups, and IGF2 expression did not vary significantly across all stages of cancer in KICH.

Immune Infiltration analysis

In this study, we used the TIMER database to correlate IGF2 gene expression and immune cell infiltration, including CD8+ T cells, CD4+ T cells, dendritic cells, macrophages, neutrophils, and purity of tumors, in three cancers: KICH, LIHC, and STAD.

**Figure 6:**
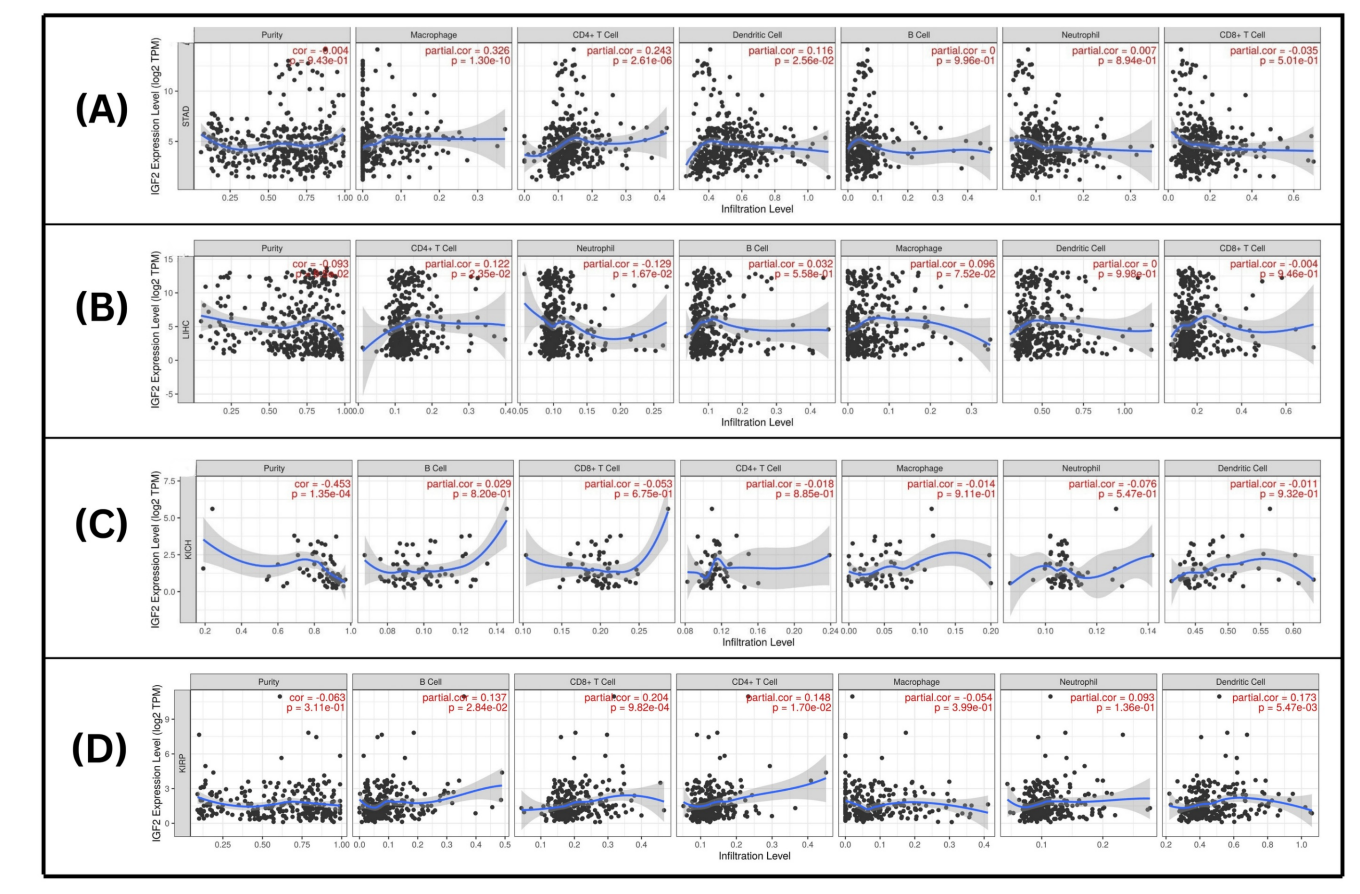
Correlation of IGF2 expression with immune cell infiltration and tumor purity in STAD, LIHC, KICH and KIRP. (A) STAD; (B) LIHC; (C) KICH; (D) KIRP STAD: Stomach adenocarcinoma; LIHC: liver hepatocellular carcinoma; KICH: kidney chromophobe; KIRP: kidney renal papillary cell carcinoma

The results demonstrated a statistically significant link between macrophages, CD4+ T cells, and dendritic cells in STAD, and between neutrophils and CD4+ T cells in LIHC. Furthermore, there was a significant association between IGF2 expression and tumor purity in KICH, but no significant correlation was observed between any of the mentioned cancers and the rest of the immune cells (P > 0.05), as shown in Figure 6.

In the context of STAD, IGF2 expression exhibited a positive correlation with macrophage infiltration, revealing a moderate correlation coefficient (Cor = 0.3260, P = 1.30E-10). Also, the levels of IGF2 expression pointed to less remarkable positive links with CD4+ T cells and dendritic cells, reflected in weak correlation coefficients of (Cor = 0.2427, 0.1158, respectively), and P-values (P = 2.61E-06, 0.0256, respectively), as shown in Figure 6A.

When looking into LIHC, the expression of IGF2 demonstrated a weak positive correlation with CD4+ T cell infiltration (Cor = 0.1220, P = 0.0235). A weak negative correlation was detected regarding IGF2 expression and the presence of neutrophils (Cor = -0.1287, P = 0.0167), as shown in Figure 6B.

In KICH, our analysis indicated a moderate negative correlation between IGF2 expression and tumor purity, with a correlation coefficient of Cor =-0.4528 (P = 0.000135), as shown in** **Figure 6C.

Analysis of KIRP revealed a weak positive correlation between IGF2 expression and the presence of B cells (correlation = 0.137, P = 0.0284), CD8+ T cells (correlation = 0.204, P = 0.000982), CD4+ T cells (correlation = 0.148, P = 0.0170), and dendritic cells (correlation = 0.173, P = 0.00547), as shown in Figure 6D.

The classification of correlation intensity was based on the following partial correlation values: (i) Moderate: values ranging from 0.4 to 0.6; (ii) Weak: values less than 0.3.

Overall survival analysis

**Figure 7:**
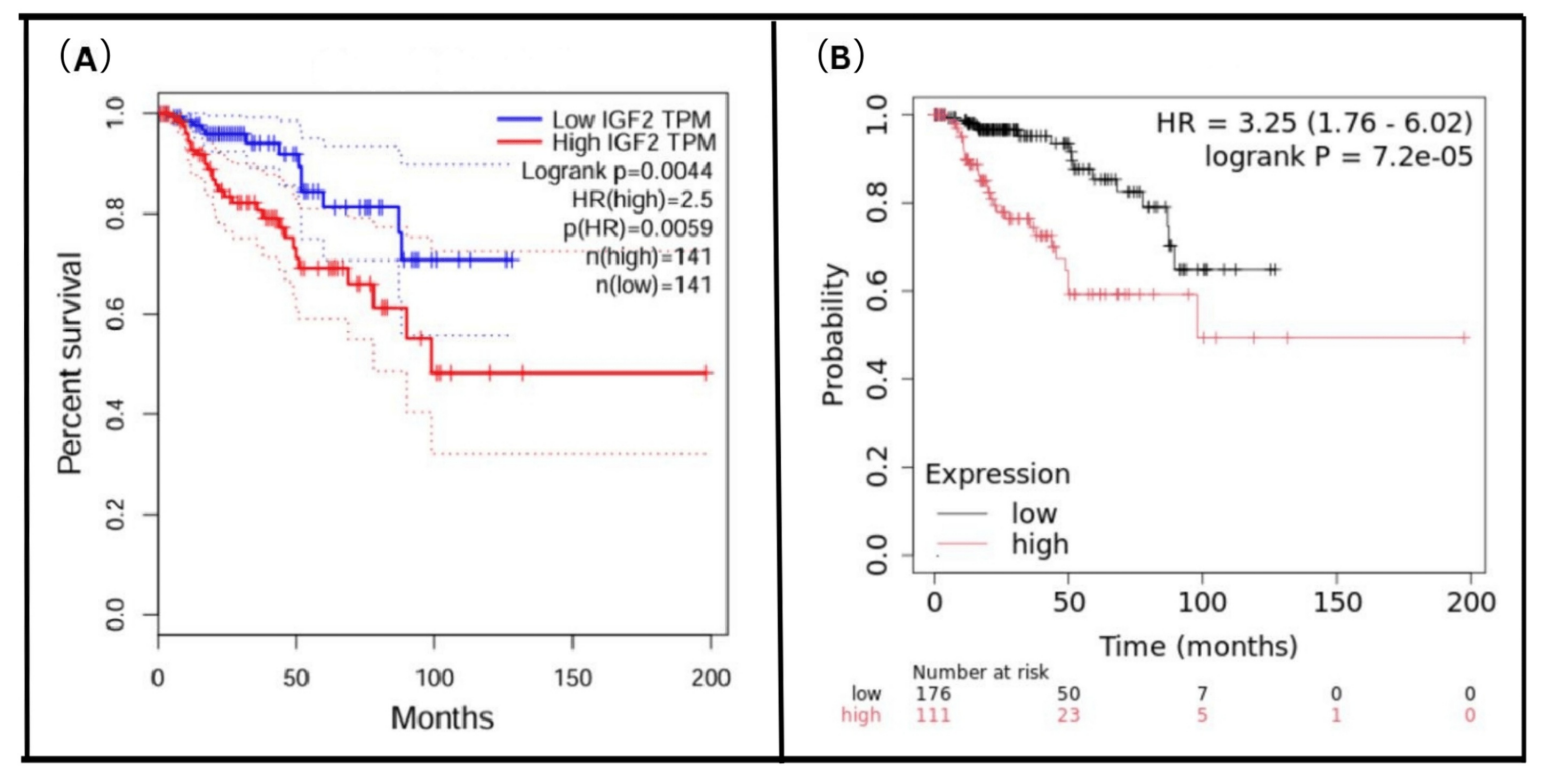
Overall survival analysis of IGF2 in KIRP cancer using GEPIA and KM plotter databases. (A) The association between IGF2 expression levels and patient survival outcomes in KIRP cancer using the GEPIA database. (B) The association between IGF2 expression levels and patient survival outcomes in KIRP cancer using the KM plotter database. IGF2: Insulin-like growth factor 2; KIRP: kidney renal papillary cell carcinoma; GEPIA: Gene Expression Profiling Interactive Analysis; KM: Kaplan-Meier

We investigated the correlation between IGF2 expression and overall survival using GEPIA and KM plotter databases. In GEPIA, overall survival was determined based on a statistical analysis with a P-value < 0.05, a 50% cutoff for both low and high expression, and a 95% confidence interval (CI). Six types of cancer showed significant correlations between overall survival and expression of the IGF2 gene: KIRC, KIRP, LIHC, mesothelioma (MESO), ovarian serous cystadenocarcinoma (OV), and uveal melanoma (UVM). Among those, one cancer: KIRP (P = 0.0044) was linked to high IGF2 expression. Five cancers: KIRC, LIHC, MESO, OV, and UVM were linked to low IGF2 expression. In KM plotter, IGF2 expression was analyzed using the mRNA seq - pan cancer. It reveals significant expression in eight cancers: KIRP, KIRC, LIHC, ovarian serous cystadenocarcinoma (OV), THCA, cervical squamous cell carcinoma, pheochromocytoma and paraganglioma (PCPG), and STAD. Two cancers, KIRP (P = 7.2e-05) and PCPG (P = 0.034), were linked to high IGF2 expression, while the other cancers were associated with low IGF2 expression. When comparing results from both databases, we found four cancers shared between the two databases: KIRC, KIRP, LIHC and ovarian serous cystadenocarcinoma (OV). One cancer, KIRP, was linked to high IGF2 expression in both databases, as shown in Figure 7.

IGF2 mutation analysis

**Figure 8:**
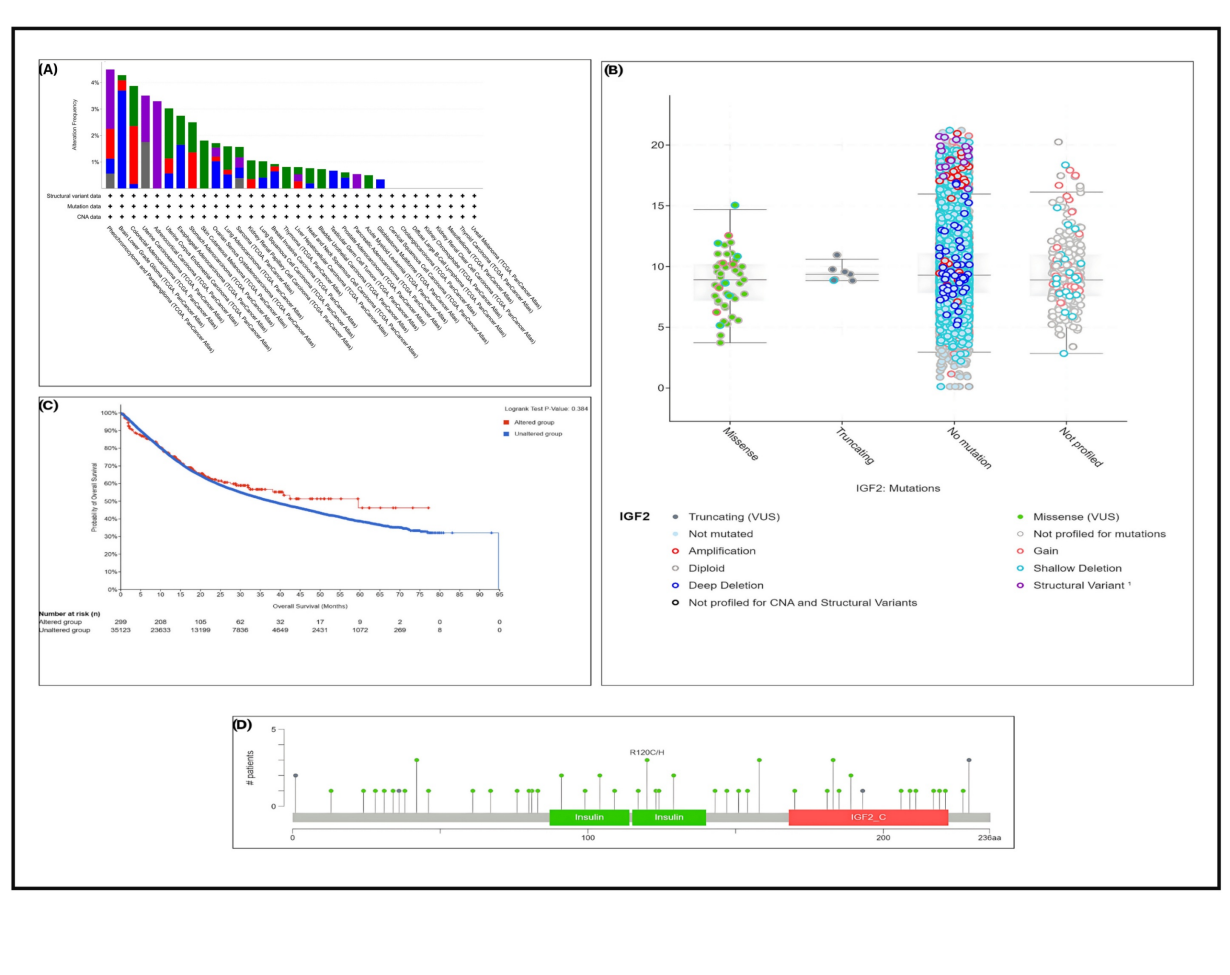
Genetic alteration analysis from the cBioPortal database. (A) The cancer type summary graph categorizes and summarizes IGF2 alterations and the distribution of mutations across different cancer types. (B) The mutation frequency plot illustrates the occurrence of driver mutations and variants of uncertain significance (VUS) in IGF2 and the frequency and position of each mutation type. (C) Alteration of IGF2 mRNA expression according to the type of mutation. (D) The survival rate among patients with various forms of cancer is correlated with genetic changes in IGF2. IGF2: Insulin-like growth factor 2

We further explored the IGF2 genetic alteration status in human cancers using the cBioPortal database, specifically focusing on data from the TCGA and Pan-Cancer Atlas cohorts, as shown in Figure 8A.Out of 10,967 patient samples, 158 (1%) exhibited a genetic alteration in IGF2.

Table 2 shows the percentages of genetic alterations for each cancer type.

**Table 2:** Percentages of genetic alterations across cancer types.

Cancer Type	Case	% with Alteration
Pheochromocytoma & Paraganglioma	178	4.49%
Brain Lower Grade Glioma	514	4.28%
Colorectal Adenocarcinoma	594	3.87%
Uterine Carcinosarcoma	57	3.51%
Adrenocortical Carcinoma	91	3.30%
Uterine Corpus Endometrial Carcinoma	529	3.02%
Esophageal Adenocarcinoma	182	2.75%
Stomach Adenocarcinoma	440	2.50%
Skin Cutaneous Melanoma	444	1.80%
Ovarian Serous Cystadenocarcinoma	584	1.71%
Lung Adenocarcinoma	566	1.59%
Sarcoma	255	1.57%
Kidney Renal Papillary Cell Carcinoma	283	1.06%
Lung Squamous Cell Carcinoma	487	1.03%
Breast Invasive Carcinoma	1084	0.92%
Thymoma	123	0.81%
Liver Hepatocellular Carcinoma	372	0.81%
Head and Neck Squamous Cell Carcinoma	523	0.76%
Bladder Urothelial Carcinoma	411	0.73%
Testicular Germ Cell Tumors	149	0.67%
Prostate Adenocarcinoma	494	0.61%
Pancreatic Adenocarcinoma	184	0.54%
Acute Myeloid Leukemia	200	0.50%
Glioblastoma Multiforme	592	0.34%

Also, we have found in the chart in Figure 8 that the IGF2 gene was altered in these types of cancers:

In STAD, the gene was altered in 2.5% of 440 cases, with mutations occurring in 1.14% (five cases) and amplifications in 1.36% (six cases). In LIHC, the gene was altered in 0.81% of 372 cases, with mutations occurring in 0.27% (one case) and structural variants in 0.27% (one case).

The plot in Figure 8B explains the frequency of each mutation type, which displays that the missense mutation was the most dominant type. The plot shows that the most frequent mutations occurred at E42D/K, R120C/H, P158L, R183C/H/S, and P229Qfs*27/E230Rfs*50.

The plot in Figure 8D displays the change in IGF2 expression according to the type of mutation. If the patient had a missense mutation, the gene expression would be higher than if they had a truncating mutation. The plot also shows that if the gene were not mutated, its expression would be at the highest level.

We have found that IGF2 was not mutated in some samples; however, patients with IGF2 mutations had a significantly worse prognosis compared to those without the mutation, with a non-significant P-value (P = 0.384), as per Figure 8C.

DNA methylation analysis

Using the UALCAN database, we analyzed IGF2 promoter methylation levels in patients with CHOL, KICH, LIHC, and KIRP.

**Figure 9:**
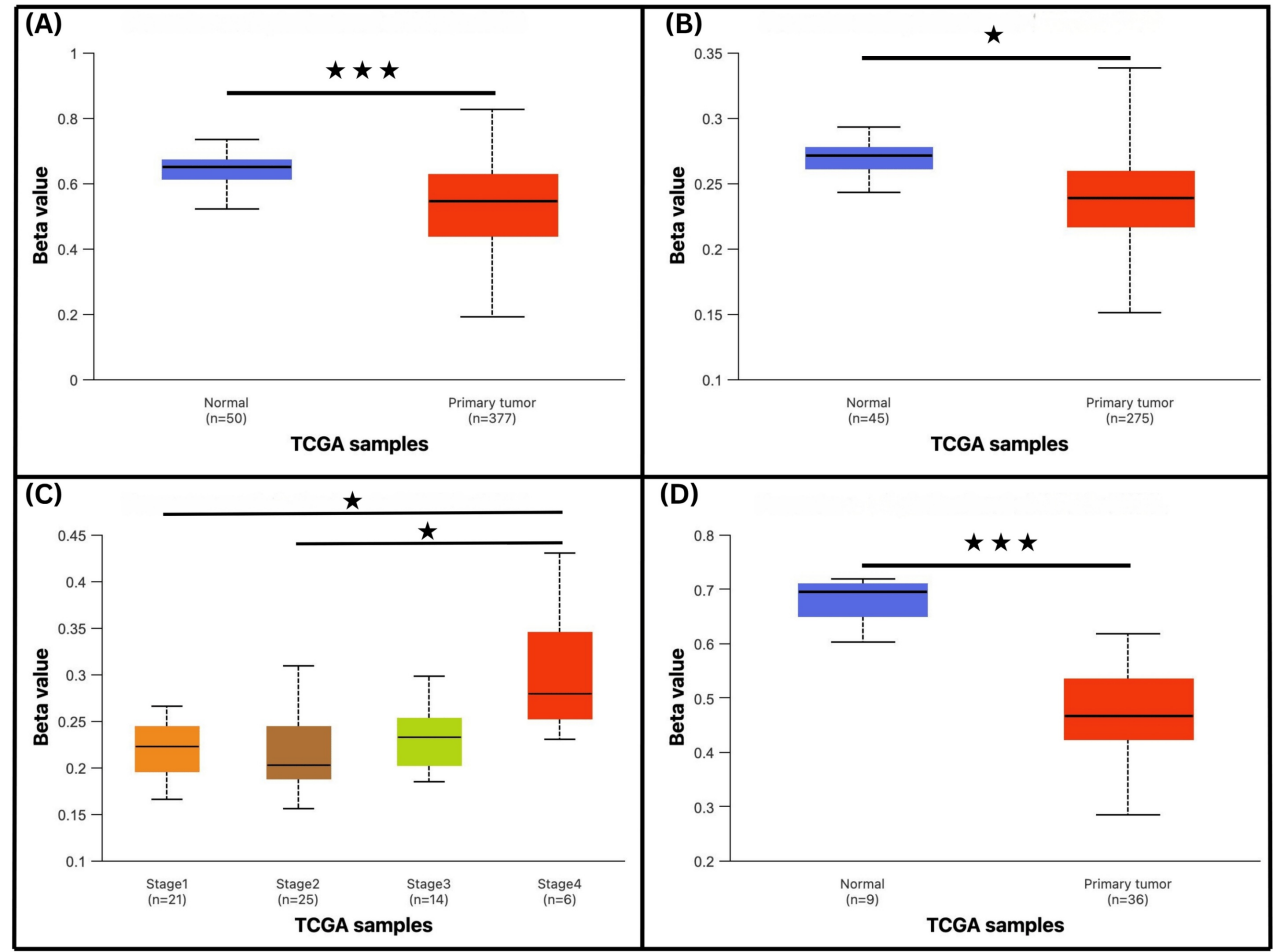
DNA methylation analysis from the UALCAN database (P-values: * < 0.05, ** <0.01, *** <0.001). (A): Promoter methylation level of IGF2 in LIHC (B): Promoter methylation level of IGF2 in KIRP (C): Promoter methylation level of IGF2 in KICH (D): Promoter methylation level of IGF2 in CHOL LIHC: Liver hepatocellular carcinoma; KICH: kidney chromophobe; CHOL: cholangiocarcinoma; KIRP: kidney renal papillary cell carcinoma

Our analysis revealed significant hypomethylation of the IGF2 promoter in LIHC (P = 1.62E-12) and KIRP (P = 1.10E-02) compared to normal tissues **​​**(Figures 9A, 9B).

In KICH, hypomethylation was observed across multiple tumor stages, with significant differences between stage 1 and stage 4 (P = 4.59E-02) and stage 2 and stage 4 (P = 4.33E-02), as shown inFigure 9C.

Conversely, we observed hypermethylation of the IGF2 promoter in CHOL (P = 1.20E-11), as presented in Figure 9D.

Gene correlation and enrichment analysis

Using the STRING and GeneMANIA databases, we examined the correlation between IGF2 and other genes in three types of cancers: CHOL, KICH, and LIHC. The results showed a positive correlation between IGF2 and several genes across these cancers.

**Figure 10:**
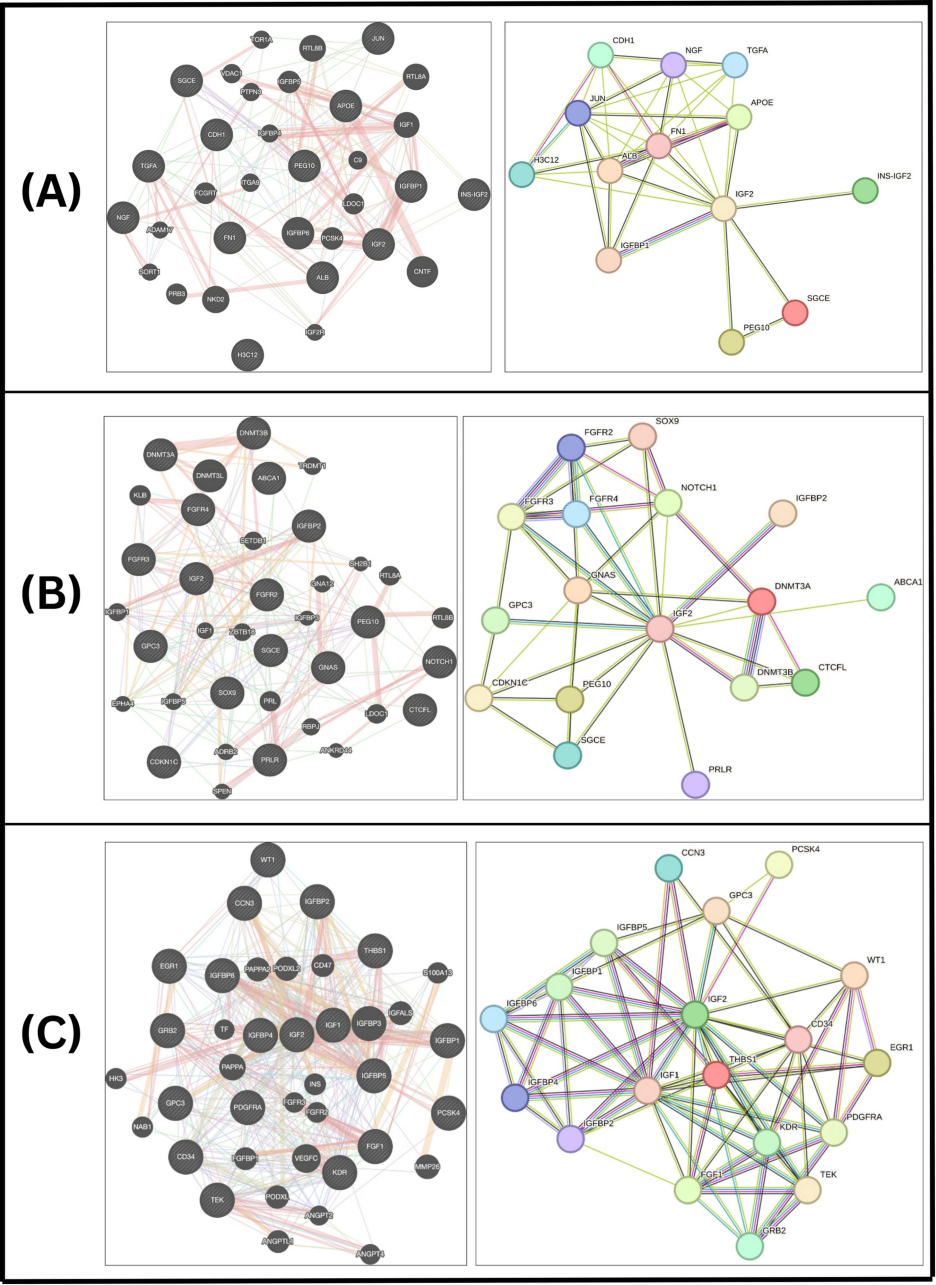
Correlation analysis of the IGF2 gene using the STRING and GeneMANIA databases. A) CHOL cancer B) LIHC cancer C) KICH cancer CHOL: Cholangiocarcinoma; LIHC: liver hepatocellular carcinoma; KICH: kidney chromophobe

For CHOL, the PPI network contained 13 nodes, with an average node degree of 5.58. There were 38 edges, with an expected number of 13 edges. The average local clustering coefficient was 0.867, and the PPI enrichment P-value was 8.84e-09. GeneMANIA’s gene interaction network indicated that IGF2 is linked to 20 potential target genes with 209 total links, 53.43% of the interactions were physical (pink/red edges), 39.72% co-expression (purple edges), 3.34% shared protein domains (yellow edges), 2.62% pathways (sky blue edges), and 0.22% genetic interactions (green edges), as shown in Figure 10A.

For LIHC, the network consisted of 17 nodes, with an average node degree of 4.71. A total of 40 edges were observed, with an expected number of 4 edges. The average local clustering coefficient was 0.806, and the PPI enrichment p-value was < 1.0e-16. GeneMANIA’s gene interaction network indicated that IGF2 is linked to 20 potential target genes with 258 total links, 34.22% of the interactions were physical (pink/red edges), 47.92% Co-expression (purple edges), 7.79% predicted (orange edges), 6.01% Co-localization (blue edges), 2.58% shared protein domains (yellow edges), and 0.29% genetic interactions (green edges), as shown in Figure 10B.

For KICH, the network included 19 nodes, with an average node degree of 7.47. There were 71 edges, with an expected number of 8 edges. The average local clustering coefficient was 0.714, and the PPI enrichment p-value was < 1.0e-16. GeneMANIA’s gene interaction network indicated that IGF2 is linked to 20 potential target genes with 683 total links, 42.10% of the interactions were physical (pink/red edges), 21.96% co-expression (purple edges), 9.80% predicted (orange edges), 9.39% pathway (sky blue edge), 11.77% shared protein domains (yellow edges), 4.97% co-localization (blue edges), and 0.01% genetic interactions (green edges), as shown in Figure 10C.

For STAD, no correlated genes were identified. PEG10 and SGCE genes were shared between CHOL and LIHC cancers. IGFBP2 and GBC3 were shared between LIHC and KICH cancers. IGFBP1 was shared between CHOL and KICH cancers.

Subsequently, we utilized the gene set (13 genes for CHOL, 17 genes for LIHC, and 19 genes for KICH) to perform GO and Kyoto Encyclopedia of Genes and Genomes (KEGG) pathway enrichment analysis using the Enrichr web tool.

**Figure 11:**
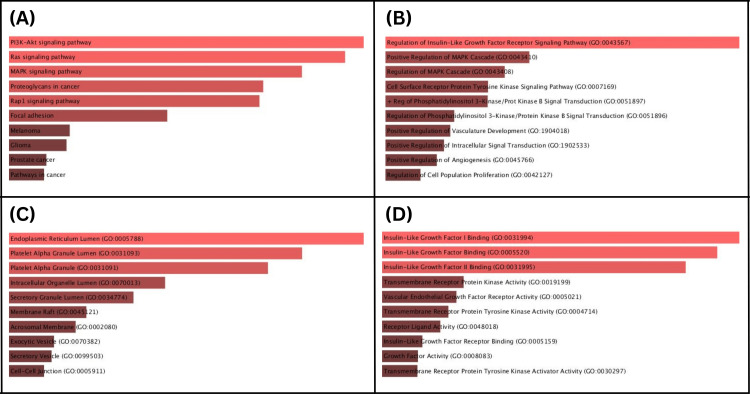
Gene ontology and pathway enrichment analysis of the KICH tumor microenvironment. (A): KICH KEGG pathway. (B): GO biological process, KICH. (C): GO Cellular component, KICH. (D): GO molecular function, KICH. KICH: Kidney chromophobe; GO: Gene Ontology; KEGG: Kyoto Encyclopedia of Genes and Genomes

In KICH, KEGG analysis revealed enrichment in the PI3K-Akt, Ras, and MAPK signaling pathways. The GO biological process was enriched in positive regulation of the insulin-like receptor signaling pathway and MAPK cascade; GO cellular component in the endoplasmic reticulum and platelet granules; and GO molecular function in insulin-like growth factor receptor binding, as shown in Figure 11.

**Figure 12:**
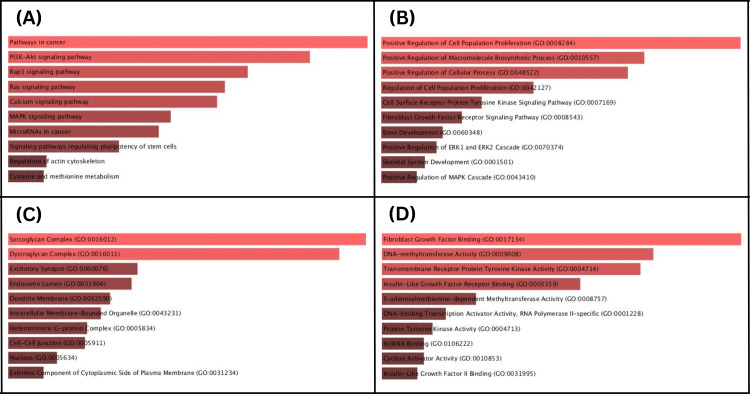
Gene ontology and pathway enrichment analysis of the LIHC tumor microenvironment. (A): KEGG pathway, LIHC (B): GO biological, LIHC (C): GO cellular, LIHC (D): GO molecular, LIHC LIHC: Liver hepatocellular carcinoma; KEGG: Kyoto Encyclopedia of Genes and Genomes; GO: Gene Ontology

For LIHC, KEGG pathways included the PI3K-Akt pathway and pathways in cancer. The GO biological process showed enrichment in regulation of cell population proliferation, the GO cellular component in sarcoglycan complex, and the GO molecular function in fibroblast growth factor binding (Figure 12).

In CHOL, KEGG enrichment highlighted pathways in cancer, the MAPK pathway. The GO biological process was enriched in regulation of cell population proliferation, GO cellular component in extracellular membrane-bound organelle, and GO molecular function in growth factor activity, as shown inFigure 13.

**Figure 13:**
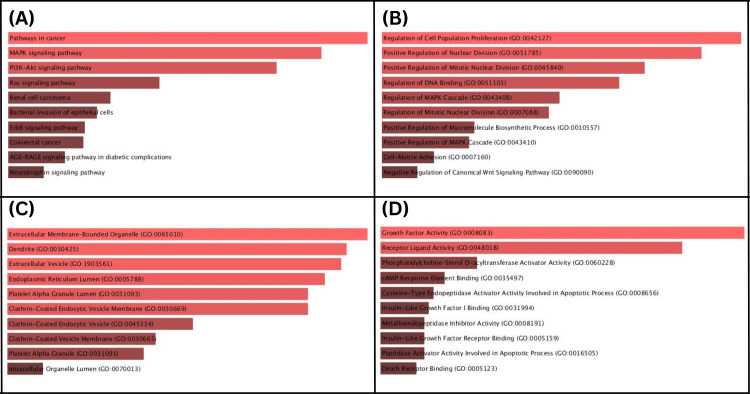
Gene ontology and pathway enrichment analysis of the CHOL tumor microenvironment. (A): KEGG Pathway, CHOL. (B): GO Biological, CHOL. (C): GO Cellular, CHOL. (D): GO Molecular, CHOL. LIHC: Liver hepatocellular carcinoma; KEGG: Kyoto Encyclopedia of Genes and Genomes; GO: Gene Ontology

Validation

After analyzing IGF2 expression in various cancers using the TIMER, GEPIA, and UALCAN databases, we validated the results (common cancers across all three databases: CHOL, KICH, LIHC, and STAD) by utilizing the Gene Expression Omnibus (GEO) database. We applied criteria of |Log2FC| > 1 and an adjusted P-value < 0.05 and then used the Julius tool to generate a volcano plot.

**Figure 14:**
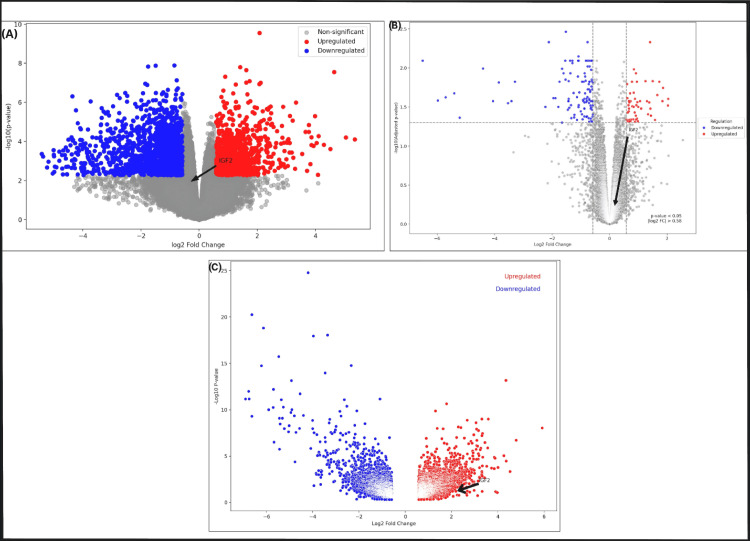
A volcano plot illustrating the expression of IGF2 in CHOL, LIHC, and STAD cancers. Red dots represent upregulated genes, blue dots indicate downregulated genes and gray dots mark genes that are not significantly expressed. (A)IGF2 expression in CHOL cancer. (B) IGF2 expression in LIHC cancer. (C) IGF2 expression in STAD cancer. IGF2: Insulin-like growth factor 2; CHOL: cholangiocarcinoma; LIHC: liver hepatocellular carcinoma; STAD: stomach adenocarcinoma

For CHOL validation in GEO, we used the GSE31370 dataset, which had a log2 fold change of 0.585, analyzing 10 samples comparing healthy and diseased individuals, as shown in Figure 14A.

For LIHC validation in GEO, the GSE263134 dataset was used, also showing a log2 fold change of 0.585, with 10 samples analyzed between healthy and diseased individuals, as shown in Figure 14B.

For STAD validation, the GSE220917 dataset was used, showing a log fold change of 0.585, with 12 samples comparing healthy and diseased individuals, as presented in Figure 14C. So far, there has been insufficient data available in GEO for KICH validation.

## Discussion

IGF2, a 7.5 kDa peptide synthesized by the liver and various tissues, has been widely linked to numerous cancers [21]. Precise regulation of the human IGF2 gene is essential for proper development and postnatal life, involving intricate genetic and epigenetic mechanisms. This regulation primarily centers around the IGF2-H19 gene cluster on Chromosome 11, influencing IGF2 expression. Disruptions in these regulatory processes can lead to increased IGF2 transcription and subsequent peptide overproduction. This overproduction can drive abnormal fetal growth and excessive cell proliferation and is implicated in the development of pediatric overgrowth syndromes and cancer [22]. The study of tumor biomarkers has gained considerable attention from scientists, with pan-cancer analysis emerging as a valuable method for discovering new tumor biomarkers [23]. Recent pan-cancer investigations have highlighted the power of large-scale multi-omics analyses in identifying prognostic and immune-related biomarkers. For example, a comprehensive study on CANT1 revealed its overexpression in multiple tumors and its association with poor survival, immune infiltration, and tumor progression across cancer types [24].

In this study, we performed pan-cancer analysis to assess the diagnostic and prognostic role of IGF2 across various cancer types. We explored IGF2 expression, prognostic importance, genetic alteration, and immune infiltration using different databases such as TIMER, GEPIA, UALCAN, cBioPortal, and KM plotter. This pan-cancer study revealed that IGF2 could be used as a diagnostic and prognostic biomarker. 

We used TIMER, GEPIA, and UALCAN to analyze the expression of IGF2 across various tumors by comparing normal and tumor samples. According to TIMER and GEPIA, IGF2 was upregulated in BRCA, CHOL, KICH, STAD, LIHC, KIRC, PRAD, KIRP, UCEC, and THCA. IGF2 expression was also analyzed using the UALCAN database. The 10 cancers identified through GEPIA and TIMER were validated by UALCAN, and the cancers common to all three databases were CHOL, KICH, LIHC, and STAD. A previous study demonstrated that IGF2 is notably upregulated in colorectal cancer, especially in CAFs, compared to normal fibroblasts [25]. Similarly, another study found that IGF2 is significantly elevated in malignant fibrous histiocytomas [26].

We used UALCAN to examine the correlation between IGF2 expression and clinicopathological factors (age, gender, race, and cancer stage) for CHOL, KICH, LIHC, and STAD. In LIHC, IGF2 expression showed significant differences based on race (Caucasians vs. African Americans, African Americans vs. Asians) and age (young vs. older adults, middle-aged vs. older adults), as well as between men and women. No significant differences were found across cancer stages. In KICH, IGF2 expression did not show any associations with age, gender, race, or cancer stages. For CHOL, IGF2 expression was significantly different based on race (Caucasians vs. African Americans) and age (middle-aged vs. older adults). In STAD, IGF2 expression varied significantly between cancer stages (stage 1 vs. stage 3) and age groups (young adults vs. middle-aged and older adults). Previous studies found no correlation between IGF2 expression and clinical parameters like age, gender, tumor location, size, or cancer stage in ovarian cancer [27,28].

This study examined the correlation between IGF2 expression and the presence of immune cells in various cancers. In gastric cancer (STAD), a moderate positive correlation with macrophages suggests that their infiltration may be linked to a worse prognosis. We also found weaker positive correlations with CD4+ T cells and dendritic cells, indicating that they contribute to the immune landscape and may influence tumor behavior and patient outcomes. Consistent with our findings on immune and stromal involvement, previous histopathological studies in breast cancer have demonstrated high IGF-II expression in both stromal and epithelial compartments. In infiltrating ductal carcinomas, IGF-II protein levels were significantly correlated with stromal proliferation, progesterone receptor (PgR), and p21 expression, suggesting that IGF-II contributes to tumor growth and differentiation through interactions with the tumor microenvironment [29]. In LIHC, a weak positive correlation between IGF2 and CD4+ T cells suggests they may still play a role in the immune response, despite the weaker association. Conversely, a weak negative correlation with neutrophils suggests that these cells may have a less prominent or inhibitory role in IGF2-driven tumorigenesis. In KICH carcinoma, a moderate negative correlation between IGF2 expression and tumor purity indicates that higher IGF2 levels are associated with lower tumor purity, suggesting a more heterogeneous tumor microenvironment with greater infiltration of non-malignant cells.

The overall survival analysis was conducted using two databases: GEPIA and KM plotter to explore the relationship between IGF2 expression and overall survival. Results from both databases indicated that higher levels of IGF2 expression were associated with better prognosis in KIRP. These findings highlight a significant link between IGF2 expression and patient survival, suggesting its potential as a prognostic biomarker for KIRP. In contrast, existing literature presents different outcomes. For instance, a study on uterine carcinosarcoma found that high IGF2 expression is linked to poorer survival [30]. Similarly, another study reported that elevated IGF2 expression predicts worse survival in ovarian cancer patients [27].

We used the cBioPortal database to investigate IGF2 mutations in various types of cancer and found that only 1% of all samples had alterations in the IGF2 gene. The most frequent types of IGF2 alterations were deep deletions and missense mutations. We also noticed that the mutation rate of IGF2 differed significantly between cancer types. In most cases, IGF2 showed little to no mutations, suggesting that it may not act as a universal cancer driver but may have a tissue-specific role. Additionally, our findings indicated that patients with IGF2 mutations had a worse prognosis compared to those without mutations in this gene.

Finally, to validate our analysis findings, we utilized data from the GEO database. GEO2R data analysis revealed that IGF2 was significantly upregulated in LIHC.

## Conclusions

This study shows that IGF2 has potential as a diagnostic biomarker for CHOL, KICH, LIHC, and STAD, and as a prognostic biomarker in KIRP. This study provides an updated integrative pan-cancer bioinformatics analysis of IGF2, offering additional insights into its expression patterns, prognostic significance, immune associations, and molecular alterations across multiple cancer types.

Importantly, IGF2 appears to play an active role in tumor biology, influencing immune cell infiltration and interacting with multiple signaling pathways. Its expression varies across cancer types and patient demographics, suggesting tissue-specific roles. These findings indicate that IGF2 could be valuable not only for early detection and prognosis but also as a potential therapeutic target. Further studies are needed to explore its mechanisms and validate its clinical applications.
